# Ferritin as a Risk Factor for Glucose Intolerance amongst Men and Women Originating from the Indian Subcontinent

**DOI:** 10.1155/2015/924387

**Published:** 2015-08-12

**Authors:** Elizabeth A. Hughes, Jeetesh V. Patel, Zosia Bredow, Paramjit S. Gill, Julia Chackathayil, Elif S. Agaoglu, Paul Flinders, Rebecca Mirrielees

**Affiliations:** ^1^University of Birmingham Centre for Cardiovascular Sciences, Sandwell and West Birmingham Hospitals NHS Trust, West Midlands B18 7QH, UK; ^2^Medical School, University of Nottingham, Nottingham NG7 2UH, UK; ^3^Primary Care Clinical Sciences, University of Birmingham, West Midlands B15 2TT, UK

## Abstract

*Background*. Serum ferritin predicts the onset of diabetes; however, this relationship is not clear amongst South Asians, a population susceptible to glucose intolerance and anaemia. *Objective*. This study tests whether ferritin levels reflect glucose tolerance in South Asians, independent of lifestyle exposures associated with Indian or British residence. *Methods*. We randomly sampled 227 Gujaratis in Britain (49.8 (14.4) years, 50% men) and 277 contemporaries living in Gujarati villages (47.6 (11.8) years, 41% men). Both groups underwent a 75 g oral-glucose-tolerance test. We evaluated lifestyle parameters with standardised questionnaires and conducted comprehensive clinical and lab measurements. *Results*. Across sites, the age-adjusted prevalence of diabetes was 9.8%. Serum ferritin was higher amongst diabetics (*P* = 0.005), irrespective of site, gender, and central obesity (*P* ≤ 0.02), and was associated with fasting and postchallenge glucose, anthropometry, blood pressure, triglycerides, and nonesterified fatty acids (*P* < 0.001). Diabetes was less in those with low ferritin (<20 mg/mL), *P* < 0.008, and risk estimate = 0.35 (95% CI 0.15–0.81), as were blood pressure and metabolic risk factors. On multivariate analysis, diabetes was independently associated with ferritin (*P* = 0.001) and age (*P* < 0.001). *Conclusion*. Ferritin levels are positively associated with glucose intolerance in our test groups, independent of gender and Indian or UK lifestyle factors.

## 1. Introduction

Iron metabolism is implicated in the incidence of diabetes and associated atherosclerotic disease [1–3]. This causative relationship is underlined by reports of an improvement of glycaemic control in response to a reduction in body iron [4, 5]. Body iron stores accumulate by dietary factors that include vitamin C and alcohol intake [6] as well as dietary iron, which in turn is a public health anxiety given that this essential nutrient influences the development of vascular disease [7].

Diabetes is particularly concentrated on the Indian subcontinent [8] whose dispersed migrant populations have a higher prevalence compared to various indigenous populations [9–14]. While studies show that established risk factors such as obesity and urbanised lifestyle “operate” amongst South Asians, it remains unclear as to how far they explain an increased susceptibility to diabetes and glucose intolerance for this group. We and others have reported the preservation of high rates of glucose intolerance amongst Indian populations both overseas and in India itself [10, 13, 14].

While levels of ferritin and other markers of body iron status are shown to be predictive of diabetes in other populations [2], their role within South Asians is not clear. Given that the improvement of body iron status to combat anaemia is a common primary healthcare concern among Indian-origin populations, [15] we determined whether serum ferritin was associated with diabetes in this group. We tested the hypothesis that serum ferritin would reflect glucose tolerance and metabolic features of diabetogenesis amongst randomly selected Gujaratis living in rural India and their contemporaries living in the UK. We examined whether the magnitude of diabetes risk from serum ferritin was independent of obesity and lifestyle factors, which included nutritional intake, physical activity, and country of residence.

## 2. Materials and Methods

Methodology of this migration study is described in more detail elsewhere [16].

### 2.1. Participants

We compared Gujarati Indians living in Sandwell (UK) who had migrated from rural villages in Navsari (Gujarat, India) with contemporaries still living in those villages of origin. Participants were randomly selected from electoral rolls (both sites). Recruitment targets were 227 participants from each country. People with high CRP and serum ferritin levels were excluded. Ethical approval for the study was obtained in Sandwell and Gujarat by the respective local research ethics committees.

### 2.2. Procedure

Participants who were invited to the clinic sessions fasted and had 75 g oral-glucose-tolerance tests (OGTT). Venous blood was collected from all participants. Blood samples were taken fasting and at 30 and 120 min after glucose challenge (based on the WHO criteria [17]).

Identical procedures for anthropometry in each site included the Leicester height measure (Seca Ltd., Birmingham, UK), weight (Seca Ltd.), waist, and hip (metal tapes). The waist was the narrowest circumference above the umbilicus and below the ribs. The hip circumference was measured over thin clothing as the widest horizontal circumference around the buttocks. Blood pressure was measured 3 times (using the mean of the last 2 for analysis), with a validated semiautomatic monitor, after >5 min sitting. Measures were rigorously standardised, with fieldworkers locally revalidated monthly, internationally every 4 months.

Dietary analysis proceeded with completion of food diaries for four consecutive days' intake and was done using WISP (Weighed Intake Software Program) version 1.23, Tinuveil software, Llanfechell, UK. Physical activity was monitored using a validated accelerometer “Caltrac” (Muscle Dynamics, Torrance, CA, USA) worn continuously for 48 hours.

### 2.3. Laboratory Methods

Details of separation, storage, and transport of blood and the analysis of lipids, lipoproteins, glucose, insulin, vitamin B12, serum folate, and C-reactive protein (CRP) are available elsewhere [16]. Venous blood collected in EDTA blood bottles was analysed for a full blood count using haematology analysers at either the Clinical Haematology department, Sandwell Hospital, UK (STAK S, Beckman Coulter Corp., Hialeah, Florida), or the Mankodi Laboratory, India (Bayer Advia, Bayer Diagnostics, Baroda, India).

Ferritin was measured on frozen serum using an automated assay on the Roche Integra 400+ (Basel, Switzerland). Given the influence of systemic inflammation on serum ferritin levels, subjects with CRP concentrations above 5 mg/L, or males with ferritin >300 ng/mL, or females with ferritin >200 ng/mL were excluded from these post hoc analyses. Anaemia was defined as a haemoglobin of <11.5 g/dL in females and <13.5 g/dL in males. Iron deficiency was defined as a ferritin measure <20 ng/mL nmol/L amongst men and women. Folate deficiency was defined as a serum folate <3 ng/mL and vitamin B12 deficiency as <200 pg/mL.

### 2.4. Power Calculation and Statistical Analysis

We hypothesised a relationship between ferritin with indices of diabetes and metabolism. For a statistically significant (*P* < 0.05, 2-sided) correlation coefficient “*r*” (at least 0.20), with a power of 80%, 193 subjects were needed. Data were analysed in SPSS v14 (SPSS Inc., Chicago, IL) using standard and nonparametric tests (as determined by Kolmogorov-Smirnov normality plots) amongst men and women. Central tendencies and variation for parametric data are presented as mean (standard deviation (SD)) or geometric means (95% CI) for nonparametric data. Comparisons were made by *t*-test, Mann Whitney test, or Chi Square tests. Univariate analysis was reported with Spearman rank correlation coefficients. Partial correlation analysis (two-tailed) was used to adjust the effects of gender and site. Linear regression models and binary logistic models were developed to test the strength of association, beta (95% CI) of potential independent predictors of glucose intolerance. The standardised beta coefficients presented allowed direct comparison (along a scale of 0-1) of the strength of each association within the model.

## 3. Results

**Figure 1:**
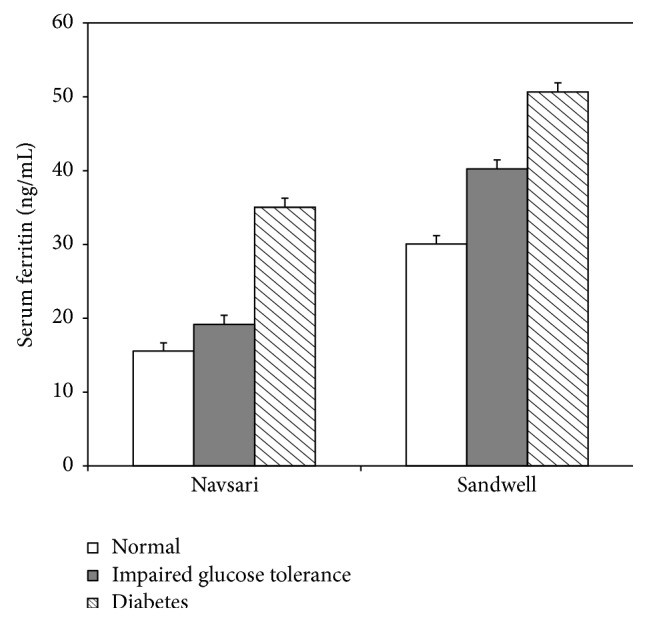
Serum ferritin in nonmigrant (Navsari) and migrant (Sandwell) Indian men and women by glucose tolerance status. Bars show geometric means (error bars show 1 standard error).

**Figure 2:**
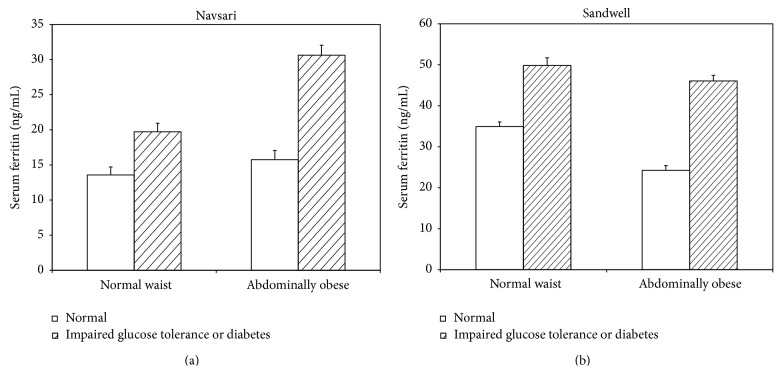
Bar chart of serum ferritin in nonmigrant (Navsari) and migrant (Sandwell) Indian Gujaratis by diabetes and central obesity status. Bars show geometric means (error bars show 1 standard error).

The percentage prevalence of diabetes (known and newly detected) was similar across sites (7.7%, and 10.7%), respectively, for India and the UK. With respect to impaired glucose tolerance, in Navsari (India) it was 20.4% and in the Sandwell (UK) it was 5.4%. Overall, the percentage of Indians with normal glucose tolerance in Navsari was 71.9% and in Sandwell it was 83.9%. Thirty-three subjects from Sandwell and 29 subjects from Navsari were excluded due to either high CRP or ferritin, leaving 114 men and 163 women from Navsari and 109 men and 118 women in Sandwell. Of subjects with high CRP or ferritin, 41% had either diabetes (all newly detected) or impaired glucose tolerance during OGTT (a higher proportion than those with normal CRP or ferritin, *P* < 0.001). Amongst subjects with normal CRP or ferritin, 9.8% had diabetes (known or newly detected during OGTT). Levels of ferritin were higher amongst diabetics (*P* = 0.005) irrespective of site (Figure 1). Levels of ferritin were higher amongst those with glucose intolerance, both in the presence (*P* = 0.04) or in the absence of central obesity (*P* = 0.02) (Figure 2). Across a gradient of deteriorating glucose tolerance (normal to impaired glucose to frank diabetes), levels of ferritin increased ordinally independently of gender and migration status (pseudo *R* ^2^ = 0.04, *P* < 0.008).

### 3.1. Ferritin Status and Diabetes Related Indices

The age-adjusted prevalence of iron deficiency was greater amongst women (*P* < 0.01) at 79.6 (72.7–86.5)% in Navsari women and 49.5 (39.6–59.4)% in Sandwell women, compared to 25.7 (17.7–33.7)% in Navsari men and 17.6 (9.8–25.3)% in Sandwell men. Measures of anthropometry and blood pressure were significantly lower amongst those with low levels of ferritin (<20 ng/mL), compared to normal ferritin, (for both men and women, Table 1). Metabolic factors such as glucose during OGTT, fasting nonesterified fatty acids (NEFA), insulin, HDL cholesterol, and triglycerides were also lower amongst those with low ferritin, significantly so for fasting triglycerides and NEFA. Diabetes was less common amongst those with low ferritin (*P* = 0.008), where the risk estimate was 0.35 (0.15–0.81).

On univariate analysis, levels of ferritin were associated with a range of metabolic factors, including fasting glucose (*r* = 0.114, *P* < 0.001), serum triglycerides (0.270, *P* < 0.001), apolipoprotein B (0.189, *P* < 0.001), insulin levels (0.113, *P* < 0.04), NEFA concentrations (0.238, *P* < 0.001), waist (0.318, *P* < 0.001) and hip circumferences (0.148, *P* < 0.001), waist to hip ratio (0.424, *P* < 0.001), and BMI (0.190, *P* < 0.001). These associations remained unchanged on separate analysis of Gujaratis at each site, except for insulin level, waist and hip circumferences, and BMI, which were no longer related to ferritin. To control confounding from central obesity, on a subgroup analysis of nonobese Gujaratis (excluding men with a waist girth ≥ 94 cm and women ≥ 80 cm), associations between ferritin and fasting glucose (*P* = 0.03), blood pressure (*P* < 0.002), and triglycerides (*P* < 0.01) were conserved. The magnitude of the relationship between ferritin and serum triglyceride levels was stronger in those with diabetes (*R* ^2^ = 0.13, *P* < 0.001) and impaired glucose tolerance (*R* ^2^ = 0.20, *P* < 0.001) compared to normal glucose (*R* ^2^ = 0.05, *P* < 0.001).

### 3.2. Anaemia amongst Indian Gujaratis

The age-adjusted prevalence of anaemia was significantly less common in Sandwell (*P* < 0.01) and greater amongst women compared to men. Rates of vitamin B12 or folate related anaemia were similar between groups. Iron deficiency anaemia was more common amongst those in Navsari, particularly the women (Table 2). Using various categories of anaemia, there was no influence of these exposures on diabetes status save iron deficiency anaemia (*P* = 0.01), which had a modest protective influence.

### 3.3. Multivariate Regression Analysis

On partial correlation analysis (controlling for the effects of gender and country of residence), levels of ferritin were positively related to total calorie intake (0.49, *P* < 0.001), energy derived from fat (0.50, *P* < 0.001), dietary iron (0.32, *P* < 0.001), and alcohol intake (0.26, *P* < 0.001) and negatively related to energy derived from carbohydrate (−0.39, *P* < 0.001). Levels of ferritin were unrelated to energy derived from protein (*P* = 0.64) and showed modest association with energy expenditure from Caltrac (0.17, *P* = 0.04) and vitamin C intake (0.16, *P* = 0.06).

On linear regression analysis, ferritin levels were independently associated with residence in India (beta (95% CI) −0.78 (−1.04–−0.52)), male gender (−1.16 (−1.38–−0.93)), anaemia (−0.434 (−0.193–−0.676)), age (0.015 (0.006–0.024)), and serum triglycerides (0.258 (0.064–0.451)) (or apolipoprotein B), all *P* < 0.001, in models that also included waist to hip ratio, fasting plasma glucose, and systolic blood pressure. Fasting glucose was also predictive of ferritin (*P* = 0.02) independently of gender, site, and waist to hip ratio, but not when measures of triglycerides or apolipoprotein B were added to regression models.

On multivariate logistic analysis, the presence of diabetes was independently associated with age (beta = 1.06 (1.02–1.10), *P* < 0.001) and serum ferritin levels (2.11 (1.34–3.12), *P* = 0.001) in a model that included migration status, gender, waist to hip ratio, anaemia, serum triglycerides, serum insulin, HDL cholesterol, and systolic blood pressure.

## 4. Discussion

Within Indian Gujarati men and women, serum ferritin, a marker of body iron status and inflammation, was consistently associated with glucose intolerance and indices of diabetes independently of lifestyle and metabolic risk factors. Serum ferritin concentrations may reflect an increased risk of diabetes amongst populations originating from the Indian subcontinent, and it is an important consideration, given the heightened prevalence of anaemia in this group.

Ferritin is an acute phase reactant, and inflammation may play a role in the inferred relationship between body iron stores and the risk of diabetes [18, 19]. Potentially, chronic inflammation is associated with diabetogenic risk factors such as obesity. However, in our study, lean Gujaratis with raised ferritin were still more likely to have diabetes than obese Gujaratis with normal ferritin, and obesity* per se* was not associated with ferritin. In a White European population, the predictive value of ferritin with respect to diabetes disappeared once other metabolic risk factors were adjusted for [2]. The cross-sectional nature of our study may limit our interpretation of an independent relationship between ferritin and glucose intolerance. Our inclusion of participants with only normal CRP and ferritin levels was an attempt to control for confounding from inflammation, but residual confounding from other inflammatory pathways or markers could persist. The measurement of soluble transferrin receptor, the expression of which is interpreted as a demand for cellular iron, is a more accurate measure of body iron status than serum ferritin alone [18]. As such, while high levels of ferritin may reflect a predisposition to diabetes in this population, the converse may also be true (a susceptibility to glucose intolerance results in higher ferritin). However, due to the cross-sectional nature of our study, the possibility of the raised serum ferritin levels caused by insulin intolerance or type 2 diabetes mellitus (reverse causation) cannot be ruled out.

We found that high levels of ferritin are associated with insulin resistance independent of lifestyle changes and gender. However, studies in other populations show that the development of type 2 diabetes mellitus is associated with dietary heme-iron intake [3, 20]. Similarly, people with a family history of diabetes exhibit high levels of ferritin irrespective of glucose tolerance status [21]. In a cross-sectional study of 12 090 Korean subjects, a positive association between serum ferritin concentration and type 2 diabetes mellitus was reported. However, they observed that raised serum ferritin concentrations were associated with type 2 diabetes mellitus, IFG, insulin resistance, and metabolic syndrome in men, yet only with IFG in women [22]. The mechanism for this discrepancy is not fully understood.

Iron is a potent metal, which could potentially interfere with insulin synthesis and secretion in the pancreas, leading to insulin resistance or reduced insulin secretion [23, 24]. For example, amongst patients with haemochromatosis undergoing OGTT, hepatic iron overload is reported to result in impaired insulin extraction [23]. While the pathophysiological mechanism is unclear, levels of triglycerides and dietary fat in this study were closely related to ferritin. A potential pathway for the intersection of body iron status and dietary fat metabolism is that ferritin can regulate the secretion of Apo B [25]. The porphyrin ring structure of Apo B allows the direct binding of ferritin proteins [26] and we are tempted to speculate that iron delivery is a normal physiological outcome of lipoprotein metabolism, which is moderated by the action of insulin. The restriction of dietary iron and fat improves pathological states such as nonalcoholic fatty liver, which in itself is a risk factor for diabetes [18]. Elsewhere, we have reported that insulin resistance is related to the suppression of NEFA during OGTT in this Gujarati Indian population [14]. In the present analysis, the relationship between ferritin and absolute measures of NEFA and insulin during OGTT was greater in magnitude than the association between ferritin and NEFA suppression. Another possible mechanism is proposed in a recent Spanish-population based cross-sectional study, suggesting that iron metabolism may contribute to the initiation of insulin resistance through inhibition of adiponectin and osteocalcin proteins involved in body glucose level regulation [27].

Our results show that when compared with the nonmigrant population (living in Gujarati villages of India), the Gujarati people who have immigrated to the UK have a higher ferritin level and less anaemia, but similar rates of diabetes. There are three potential reasons for this. Firstly, the migration to the UK is more likely to involve a greater likelihood of nonvegetarianism, as opposed to the traditional Gujarati vegetarian diet. Hence, the prevalence of anaemia reflects dietary changes. Secondly, a nonspecific marker like ferritin could be linked to the chronic inflammatory states that are associated with obesity, which would also be exacerbated by dietary changes. Finally, the changes in prevalence could be due to a higher availability of medical resources in Sandwell (compared to Gujarati villages), so more routine blood counts have been undertaken. This may therefore result in more patients being treated for anaemia diagnosed on the serum results rather than on clinical presentation alone, thus more ferrous sulphate prescribed to the population. This would result in a higher serum ferritin and lower prevalence of anaemia in this group of people.

The implication of these findings is that the interpretation of serum ferritin amongst people from the Indian subcontinent is an important concern, not only for the treatment of anaemia [13] but also for the paradoxical risk of diabetes. According to a study done on *β*-thalassaemia major patients, which investigated the effects of chelation therapy on glucose levels, treating patients with deferoxamine and deferiprone (combined chelation for lowering ferritin levels) resulted in an improvement of glucose metabolism disorders in *β*-thalassaemia major patients [28]. The increased risk of diabetes is potentially a consideration for clinician treating particular ethnic groups with iron replacement therapy. This study raises concerns as to whether reference ranges for serum ferritin in South Asians are sensitive to a potential risk of diabetes. However, the present study design would be unsuitable and underpowered to answer such questions. In summary, levels of ferritin are associated with diabetes and metabolic factors amongst Indian Gujaratis, and this relationship is independent of site and gender and remains independent of the effects on obesity. Further work is warranted to determine the prognostic and diagnostic implications of body iron status within this population.

## Figures and Tables

**Table 1:** Diabetes related indices by body iron status amongst Indian Gujaratis.

	Men		Women	
	Normal ferritin (≥20 and <300 ng/mL) *n* = 135 (67 migrants)	Low ferritin (<20 ng/mL) *n* = 45 (16 migrants)	Normal ferritin (≥20 and <300 ng/mL) *n* = 62 (44 migrants)	Low ferritin (<20 ng/mL) *n* = 136 (52 migrants)
Age (years)	48.7 (46.5–50.9)	50.0 (45.6–54.5)	50.9 (47.9–53.9)	47.3 (45.2–49.5)
Body mass index (kg/m^2^)^#^	23.5 (22.8–24.3)	21.4 (20.1–22.7)	24.6 (23.5–25.8)	22.4 (21.6–23.2)
Waist circumference (cm)	86.0 (83.6–88.5)	81.3 (77.7–85.0)	80.0 (77.3–82.6)	74.6 (72.4–76.7)
Hip circumference (cm)	95.9 (94.4–97.3)	93.2 (90.4–96.0)	97.5 (95.0–99.9)	94.3 (92.4–96.1)
Systolic blood pressure (mmHg)^#^	130 (126–133)	120 (114–126)	122 (116–129)	113 (109–116)
Diastolic blood pressure (mmHg)^#^	80.1 (78.1–82.2)	73.6 (70.3–76.9)	74.6 (71.5–77.8)	69.8 (68.1–71.5)
Fasting glucose (mmol/L)	5.64 (5.31–5.97)	5.30 (4.53–6.07)	5.33 (4.91–5.75)	5.05 (4.89–5.22)
2 hr glucose (mmol/L)	5.91 (5.56–6.26)	5.82 (5.31–6.34)	6.15 (5.68–6.62)	5.88 (5.56–6.20)
Fasting nonesterified fatty acids (mmol/L)	0.39 (0.35–0.42)	0.39 (0.31–0.47)	0.41 (0.36–0.47)	0.30 (0.26–0.33)
Fasting triglycerides (mmol/L)^*∗*#^	1.08 (1.00–1.17)	0.90 (0.78–1.04)	1.08 (0.98–1.19)	0.87 (0.81–0.93)
Apolipoprotein B to AI ratio	0.727 (0.689–0.764)	0.759 (0.669–0.850)	0.705 (0.667–0.743)	0.652 (0.621–0.684)
HDL cholesterol (mmol/L)	1.23 (1.17–1.29)	1.20 (1.10–1.31)	1.27 (1.19–1.36)	1.18 (1.13–1.23)

Data are mean (95% CI) or ^*∗*^geometric mean (95% CI). ^#^Differences are significant for body mass index (0.008), triglycerides (<0.05), systolic (0.006), and diastolic blood pressure (0.001).

**Table 2:** Age-adjusted prevalence of anaemia amongst Indian Gujaratis by residence and gender.

	Navsari men	Sandwell men	Navsari women	Sandwell women
All anaemia	29.7 (22.1–37.2)	16.1 (9.1–20.2)	45.1 (37.5–52.7)	20.2 (12.8–27.5)
Vitamin B12 or folate deficiency anaemia	7.6 (3.2–12.0)	6.9 (2.3–11.5)	14.5 (9.2–19.9)	3.7 (0.3–7.2)
Iron deficiency anaemia	11.3 (6.1–16.6)	3.0 (0–6.2)	30.0 (22.4–37.6)	13.2 (7.0–19.3)

Data are percent (95% CI).
